# Supplementary material for: Prognostic Factors for Functional Outcome of Patients with Optic Nerve Sheath Meningiomas Treated with Stereotactic Radiotherapy–Evaluation of Own and Meta-Analysis of Published Data
Source: Cancers (Basel). 2021 Jan 29;13(3):522. doi: 10.3390/cancers13030522 (PMC7866383; doi:10.3390/cancers13030522)
Supplement: Supplementary file 1 [file cancers-13-00522-s001.pdf]

Prognostic Factors for Functional Outcome of Patients with Optic Nerve Sheath Meningiomas Treated with Stereotactic Radiotherapy–Evaluation of Own and Meta-Analysis of Published Data

Bogdan Pintea, Azize Boström, Sotiris Katsigiannis, Konstantinos Gousias, Rares Pintea, Brigitta Baumert and Jan Boström

Table S1. Clinical data of the MediClin Robert Janker Clinic patients.

Patient	FU Years	Gender Male = 1, Female = 0	Intracranial Site yes = 1, no = 0	Anterior = 0 vs Posterior = 1	Tumorvolume PTV (ccm)	Fractions (n)	GD (Gy)	Retinadosis (Gy)	Age (years)	Visus ill eye	Improve = 1, no improve = 0
patient 1	1	0	1	1	1.52	28	50.4	39.0	39	0.32	1
patient 2	3	1	0	0	1.22	28	50.4	43.6	46	0.8	1
patient 3	3	0	0	0	1.65	28	51.33	44.9	21	0.5	1
patient 4	4	1	1	1	1.71	28	50.4	12.2	36	0.4	1
patient 5	1	1	0	0	1.90	28	50.4	49.8	55	0.8	1
patient 6	3	0	1	1	3.76	28	50.4	50.5	57	0.4	1
patient 7	2	0	0	0	2.22	30	51.56	49.9	49	0.4	1
patient 8	2	0	1	1	2.10	28	50.4	26.6	50		1
patient 9	1	1	0	0	1.90	28	50.4	42.2	54		0
patient 10	5	0	1	1	2.63	28	50.4	46.2	49	0.1	0
patient 11	4	1	1	1	2.61	28	50.4	43.6	67	0.4	0
patient 12	5	1	1	1	0.80	1	14	10.7	19	0.02	0
patient 13	1	0	1	1	1.64	30	50.4	51.6	47	0.05	0

Table S2. ROC analysis of the published ONSM patients data.

**ROC curve**	
Variable	Visus_ill_eye
Classification variable	Improve = 1 vs no improve = 0
Sample size	100
Positive group [a]	68 (68 %)
Negative group [b]	32 (32 %)
[a] Improve 1 = 1, [b] no improve 0 = 0	
Improve prevalence (%)	68
**Area under the ROC curve (AUC)**	
Area under the ROC curve (AUC)	0.617
Standard Error [a]	0.0642
95% Confidence interval [b]	0.491 to 0.742
z statistic	1.819
Significance level P (Area = 0.5)	0.0689
[a] DeLong et al., 1988; [b] AUC ± 1.96 SE	
**Youden index**	
Youden index J	0.2574
95% Confidence interval [a]	0.1172 to 0.3896
Associated criterion	>0.05
95% Confidence interval [a]	>0.017 to >0.28
Sensitivity	88.24
Specificity	37.50
[a] BCa bootstrap confidence interval (1000 iterations; random number seed: 978).	
**Optimal criterion**	
Optimal criterion [a]	>0.28
95% Confidence interval [b]	>0.02 to >0.75
Sensitivity	75.00
Specificity	50.00

[a] Taking into account disease prevalence (68,0%) and estimated costs: cost False Positive: 10; cost False Negative: 4; cost True Positive: 0; cost True Negative: 0
[b] BCa bootstrap confidence interval (1000 iterations; random number seed: 978).
